# The biological effects of higher and lower positive end-expiratory pressure in pulmonary and extrapulmonary acute lung injury with intra-abdominal hypertension

**DOI:** 10.1186/cc13920

**Published:** 2014-06-13

**Authors:** Cíntia Lourenco Santos, Lillian Moraes, Raquel Souza Santos, Cynthia dos Santos Samary, Johnatas Dutra Silva, Marcelo Marcos Morales, Vera Lucia Capelozzi, Marcelo Gama de Abreu, Alberto Schanaider, Pedro Leme Silva, Cristiane Sousa Nascimento Baez Garcia, Paolo Pelosi, Patricia Rieken Macedo Rocco

**Affiliations:** 1 Laboratory of Pulmonary Investigation, Carlos Chagas Filho Biophysics Institute, Federal University of Rio de Janeiro, Av. Carlos Chagas Filho, s/n, Bloco G-014, Ilha do Fundão, 21941-902 Rio de Janeiro, RJ, Brazil; 2 Laboratory of Experimental Surgery, Faculty of Medicine, Federal University of Rio de Janeiro, Av. Professor Rodolpho Paulo Rocco, 225, Ilha do Fundão, 21941-913 Rio de Janeiro, RJ, Brazil; 3 Laboratory of Cellular and Molecular Physiology, Carlos Chagas Filho Biophysics Institute, Federal University of Rio de Janeiro, Av. Carlos Chagas Filho, s/n, Bloco G2-048, Ilha do Fundão, 21941-902 Rio de Janeiro, RJ, Brazil; 4 Department of Pathology, School of Medicine, University of São Paulo, Av. Doutor Arnaldo, 455, 01246-903 São Paulo, SP, Brazil; 5 Pulmonary Engineering Group, Department of Anesthesiology and Intensive Care Therapy, University Hospital Carl Gustav Carus, Dresden University of Technology, Fetschertsrasse 74, 01307 Dresden, Germany; 6 Rio de Janeiro Federal Institute of Education, Science and Technology, Rua Carlos Wenceslau, nº 343, Realengo, 21715-000 Rio de Janeiro, RJ, Brazil; 7 IRCCS AOU San Martino-IST, Department of Surgical Sciences and Integrated Diagnostics, University of Genoa, Largo Rosanna Benzi 8, 16132 Genoa, Italy

## Abstract

**Introduction:**

Mechanical ventilation with high positive end-expiratory pressure (PEEP) has been used in patients with acute respiratory distress syndrome (ARDS) and intra-abdominal hypertension (IAH), but the role of PEEP in minimizing lung injury remains controversial. We hypothesized that in the presence of acute lung injury (ALI) with IAH: 1) higher PEEP levels improve pulmonary morphofunction and minimize lung injury; and 2) the biological effects of higher PEEP are more effective in extrapulmonary (exp) than pulmonary (p) ALI.

**Methods:**

In 48 adult male Wistar rats, ALIp and ALIexp were induced by *Escherichia coli* lipopolysaccharide intratracheally and intraperitoneally, respectively. After 24 hours, animals were anesthetized and mechanically ventilated (tidal volume of 6 mL/kg). IAH (15 mmHg) was induced and rats randomly assigned to PEEP of 5 (PEEP5), 7 (PEEP7) or 10 (PEEP10) cmH_2_O for 1 hour.

**Results:**

In both ALIp and ALIexp, higher PEEP levels improved oxygenation. PEEP10 increased alveolar hyperinflation and epithelial cell damage compared to PEEP5, independent of ALI etiology. In ALIp, PEEP7 and PEEP10 increased lung elastance compared to PEEP5 (4.3 ± 0.7 and 4.3 ± 0.9 versus 3.1 ± 0.3 cmH_2_O/mL, respectively, *P* <0.01), without changes in alveolar collapse, interleukin-6, caspase-3, type III procollagen, receptor for advanced glycation end-products, and vascular cell adhesion molecule-1 expressions. Moreover, PEEP10 increased diaphragmatic injury compared to PEEP5. In ALIexp, PEEP7 decreased lung elastance and alveolar collapse compared to PEEP5 (2.3 ± 0.5 versus 3.6 ± 0.7 cmH_2_O/mL, *P* <0.02, and 27.2 (24.7 to 36.8) versus 44.2 (39.7 to 56.9)%, *P* <0.05, respectively), while PEEP7 and PEEP10 increased interleukin-6 and type III procollagen expressions, as well as type II epithelial cell damage compared to PEEP5.

**Conclusions:**

In the current models of ALI with IAH, in contrast to our primary hypothesis, higher PEEP is more effective in ALIp than ALIexp as demonstrated by the activation of biological markers. Therefore, higher PEEP should be used cautiously in the presence of IAH and ALI, mainly in ALIexp.

## Introduction

The optimization of positive end-expiratory pressure (PEEP) plays a relevant role in preventing the development of ventilator-induced acute lung injury (VALI) in patients with acute respiratory distress syndrome (ARDS) [1]. The level of PEEP required to avoid alveolar derecruitment and possibly atelectrauma is likely higher during intra-abdominal hypertension (IAH), compared to normal intra-abdominal pressure. Experimental and clinical studies have suggested that the etiology of ARDS (pulmonary and extra-pulmonary, ALIp and ALIexp, respectively) may also affect the response to PEEP. According to those studies, in ALIp alveolar edema and tissue consolidation predominate, whereas ALIexp is associated with potentially recruitable alveolar collapse [2]. Nevertheless, previous studies in small animals [3], large animals [4] and in patients [5,6] showed that PEEP represents a compromise. If PEEP is too low, cyclic recruitment/derecruitment of the lung during mechanical tidal breath may occur, leading to shear stress [7]. Conversely, high PEEP used to keep lungs open may overdistend certain lung regions, resulting in increased lung injury.

In view of these facts, we aimed to investigate the effects of different PEEP levels on arterial blood gases, lung mechanics and histology, as well as to identify biological markers associated with inflammation, apoptosis, fibrogenesis and damage inflicted to alveolar epithelial and endothelial cells in models of ALIp and ALIexp with IAH in rats. We hypothesized that: 1) higher PEEP levels improve pulmonary morphofunction and minimize lung injury; and 2) the biological effects of higher PEEP are more effective in extrapulmonary (exp) than in pulmonary (p) ALI.

## Materials and methods

This study was approved by the Animal Research Ethics Committee of the Federal University of Rio de Janeiro Health Sciences Center (CEUA-CCS, 019). All animals received humane care in compliance with the ‘Principles of Laboratory Animal Care’ formulated by the National Society for Medical Research and the ‘Guide for the Care and Use of Laboratory Animals’ prepared by the U.S. National Academy of Sciences.

### Animal preparation and experimental protocol

Forty-eight Wistar rats (300 to 350 g) were submitted to the following sequence of events: 1) random assignment to receive *Escherichia coli* lipopolysaccharide (LPS) O55:B5 (Sigma Chemical Co., St. Louis, MO, USA) either intratracheally (i.t.) (200 μg) (pulmonary ALI (ALIp), n = 24), or intraperitoneally (i.p.) (1,000 μg) (extrapulmonary ALI (ALIexp), n = 24), suspended in saline solution with total volumes equal to 200 μL and 1,000 μL, respectively. In our experience, these doses of *E.c coli* LPS yield a similar 1.5-fold-increase in static lung elastance (Est,L) in ALIp and ALIexp [8,9]; 2) waiting period of 24 hours for development of lung injury; 3) sedation (10 mg/kg i.p. diazepam, Compaz®, Cristália, Itapira, SP, Brazil), general anesthesia (100 mg/kg i.p. ketamine, Ketamin-S + ®, Cristália, Itapira, SP, Brazil, and 10 mg/kg i.p. xylazine, Rompun 2%, Bayer®, São Paulo, Brazil), subcutaneous local anesthesia (Xylestesin® 2%, Cristália, Itapira, Brazil), tracheostomy and mechanical ventilation; 4) induction of IAH; 5) random assignment to different levels of PEEP, namely 5, 7 or 10 cmH_2_O (PEEP5, PEEP7, PEEP10, respectively) (n = 8 per PEEP level in each ALI group). In small animals, PEEP5 likely corresponds to ‘moderate PEEP’, whereas PEEP7 and PEEP10 correspond to ‘higher PEEP’ [3,10].

Anesthetized animals were kept in the supine position. After median neck incision, a polyethylene catheter (PE-50) was introduced into the right internal carotid artery for blood sampling and mean arterial blood pressure (MAP) measurement. Heart rate (HR), MAP, and rectal temperature were continuously recorded (Networked Multiparameter Veterinary Monitor LifeWindow 6000 V, Digicare Animal Health, Boynton Beach, FL, USA). Body temperature was maintained at 37.5 ± 1°C using a heating blanket. Administration of lactated ringer solution (10 mL/kg/hour) was performed via the tail vein to keep fluid homeostasis. Gelafundin® (B. Braun, São Gonçalo, RJ, Brazil) was administered (in steps of 0.5 mL) to maintain MAP >70 mmHg.

Animals were paralyzed (2 mg/kg intravenous vecuronium bromide – Vecuron, Cristália, Itapira, SP, Brazil) and mechanically ventilated (Servo-i, MAQUET, Solna, Sweden) in volume-controlled mode with: V_T_ = 6 mL/kg, respiratory rate (RR) = 80 breaths/min, fraction of inspired oxygen (FIO_2_) = 1.0, and zero end-expiratory pressure (ZEEP) for five minutes in order to evaluate if the degree of lung damage was similar in ALIp and ALIexp before inducing IAH, while avoiding recruitment by PEEP. Lung mechanics were measured (Baseline-ZEEP). Arterial blood (300 μL) was drawn into a heparinized syringe to determine arterial oxygen partial pressure (PaO_2_) (i-STAT, Abbott Laboratories, Abbott Park, Illinois, USA) (Baseline-ZEEP). After blood gas analysis, functional data (MAP and rectal temperature) were collected.

**Figure 1:**
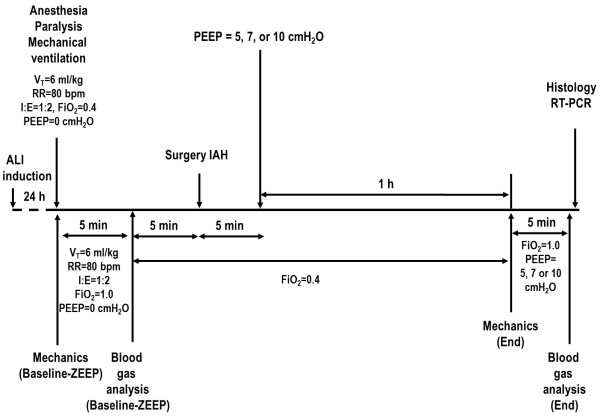
**Timeline representation of the experimental protocol.** ALI: acute lung injury; FIO_2_: fraction of inspired oxygen; IAH: intra-abdominal hypertension; I:E: inspiratory-to-expiratory ratio; PEEP: positive end-expiratory pressure; RR: respiratory rate; RT-PCR: real-time reverse transcription polymerase chain reaction; V_T_: tidal volume; ZEEP: zero end-expiratory pressure.

In order to induce IAH, a midline laparotomy (3-cm incision) was performed to expose the abdominal cavity, and 15-cm hydrophilic gauze compresses (Cremer, Blumenau, SC, Brazil) were placed in the four quadrants (one gauze/quadrant). A catheter (PE-240) was inserted into the peritoneum for continuous intra-abdominal pressure (IAP) measurement [9], and a 2–0 silk suture was used to tie the catheter in place and ensure that there was no leaking. Both layers of the abdominal cavity were closed with 3–0 monofilament nylon suture (Ethilon®, São Paulo, SP, Brazil), which was tightened to maintain IAP of 15 mmHg [9]. IAP was maintained at this level throughout the experiment. Lung mechanics were measured after a one-hour ventilation period (End). Following this step, FiO_2_ was set at 1.0 and PaO_2_ measured after five minutes. Animals were then killed with intravenous sodium thiopental 60 mg/kg, and their lungs extracted for histological and molecular biology analysis (Figure 1).

### Data acquisition and processing

Airflow, airway (P,aw) and esophageal pressures (Pes) were continuously recorded throughout the experiments with a computer running custom software written in LabVIEW® (National Instruments, Austin, TX, USA) [11]. V_T_ was calculated by digital integration of the flow signal. Changes in esophageal pressure (ΔPes), which reflect changes in chest wall pressure, were measured with a 30-cm long water filled catheter (PE205) with side holes at the tip connected to a SCIREQ differential pressure transducer (UT-PL-400, SCIREQ, Montreal, QC, Canada). The catheter was passed into the stomach and then slowly returned into the esophagus. Its proper positioning was assessed using the occlusion test described elsewhere [12]. Transpulmonary pressure (P,L) was calculated during inspiration and expiration as the difference between airway and esophageal pressures [8,9]. All signals were filtered (100 Hz), amplified in a 4-channel signal conditioner (SC-24, SCIREQ, Montreal, QC, Canada) and sampled at 200 Hz with a 12-bit analogue-to-digital converter (National Instruments). Est,L was computed off-line by a routine written in MATLAB (Version R2007a; The Mathworks Inc, Natik, MA, USA) [8] and calculated as the difference between P,L at end-inspiration and end-expiration (five second occlusion maneuvers) divided by V_T_.

### Histology

#### Light microscopy

A laparotomy was performed at the end of the experiments (End). Heparin (1,000 IU) was injected intravenously in the tail vein. The trachea was clamped at the respective PEEP level to avoid loss of end-expiratory lung volume, and lungs were carefully removed *en bloc*, fixed in 3% buffered formaldehyde, paraffin embedded, and stained with hematoxylin-eosin. Lung morphometric analysis was performed using an integrating eyepiece with a coherent system consisting of a grid with 100 points and 50 lines (known length) coupled to a conventional light microscope (Olympus BX51, Olympus Latin America-Inc., São Paulo, SP, Brazil). The volume fractions of the lung occupied by collapsed alveoli or hyperinflated structures (alveolar ducts, alveolar sacs or alveoli; maximum chord length in air >120 μm) were determined by the point-counting technique [13] at a magnification of x200 across 10 random, non-coincident microscopic fields.

#### Transmission electron microscopy

Three slices measuring 2 × 2 × 2 mm were cut from three different segments of the right lung and fixed (2.5% glutaraldehyde and 0.1 M phosphate buffer (pH = 7.4)) for transmission electron microscopy (TEM) (JEOL 1010 Transmission Electron Microscope, Tokyo, Japan). Each TEM image (20 per animal) was analyzed for damage to type I and II epithelial cells and endothelial cells at three different magnifications. Pathologic findings were graded according to a 5-point semi-quantitative severity-based scoring system as: 0 = normal lung parenchyma, 1 = changes in 1% to 25%, 2 = changes in 26% to 50%, 3 = changes in 51% to 75%, and 4 = changes in 76% to 100% of the examined tissue [3,8]. The following aspects were assessed on TEM of diaphragm muscle: 1) myofibril abnormalities, defined as disruption of myofibril bundles or disorganized myofibrillar pattern with edema of the Z disc; 2) mitochondrial injury with abnormal, swollen mitochondria and abnormal cristae; and 3) miscellaneous, which included lipid droplets, vacuoles, inter-myofibril space and nuclei. The pathologic findings were graded according to a 5-point semiquantitative severity-based scoring system, as follows: 0 = normal lung parenchyma or diaphragm, 1 = changes in 1% to 25%, 2 = changes in 26% to 50%, 3 = changes in 51% to 75%, and 4 = changes in 76% to 100% of the examined tissue. The pathologist or technician working on the light microscopy and TEM images was blinded to group assignment.

#### Biological markers of inflammation, apoptosis, fibrogenesis, and lung epithelial and endothelial cell damage

Quantitative real-time reverse transcription polymerase chain reaction (RT-PCR) was performed to measure biological markers associated with inflammation (interleukin (IL)-6), apoptosis (caspase-3), fibrogenesis (type III procollagen (PCIII)), and damage inflicted on alveolar type I (receptor for advanced glycation end-products (RAGE)), and endothelium (vascular cell adhesion molecule-1 (VCAM-1)). Central slices of right lung were cut, collected in cryotubes, quick-frozen by immersion in liquid nitrogen, and stored at −80°C. Total RNA was extracted from frozen tissues using the SV Total RNA Isolation System (Promega Corporation, Fitchburg, WI, USA) following the manufacturer’s recommendations. RNA concentration was measured by spectrophotometry in Nanodrop ND- 1000. First-strand cDNA was synthesized from total RNA using GoTaq® 2-STEP RT qPCR System (Promega). The following primers (Integrated DNA Technologies, San Diego, CA, USA) were used: IL-6, caspase-3, PCIII, RAGE, VCAM-1 and acidic ribosomal phosphoprotein P0, 36B4. Relative mRNA levels were measured with a SYBR green detection system using ABI 7500 real-time PCR (Applied Biosystems, Foster City, CA, USA). Samples were measured in triplicate. For each sample, the expression of each gene was normalized to housekeeping gene expression (acidic ribosomal phosphoprotein P0, 36B4) and expressed as fold changes relative to PEEP5, using the 2^–ΔΔCt^ method, where ΔCt = Ct, reference gene – Ct, target gene. An additional file shows in more detail the description of the primers used in the experiments [see Additional file 1].

### Statistical analysis

Differences between different levels of PEEP were assessed by one-way analysis of variance (ANOVA) followed by Tukey’s test for each ALI etiology. MAP (Baseline-ZEEP up to End) was compared by one-way ANOVA for repeated measures followed by Bonferroni’s test. Parametric data were expressed as mean ± SD and nonparametric data as median (interquartile range). All tests were performed using GraphPad Prism v5.00 statistical software package (GraphPad Software, La Jolla, CA, USA). Significance was established at *P* <0.05.

## Results

**Figure 2:**
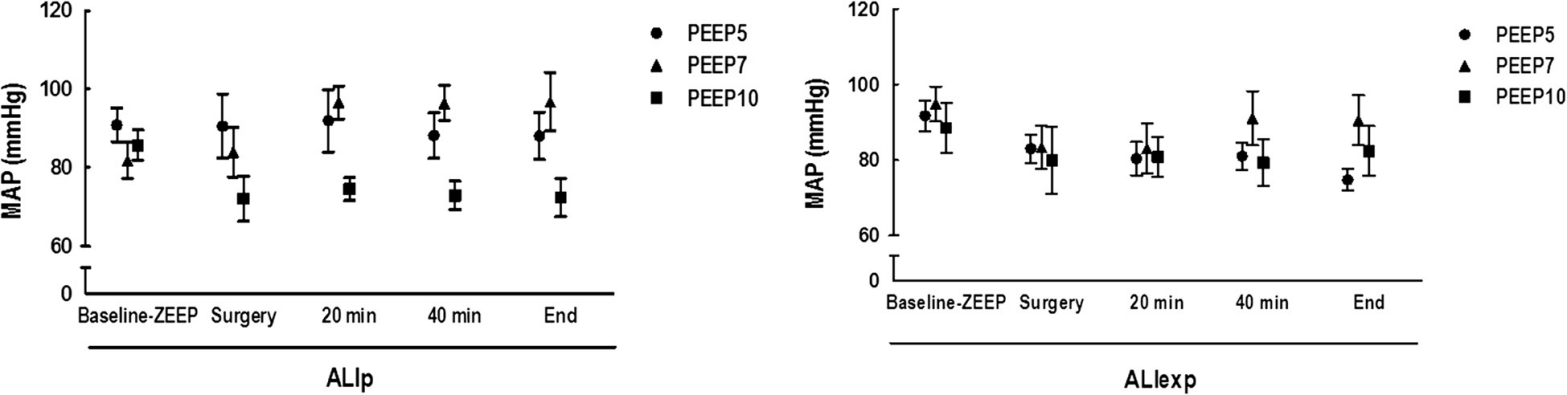
**MAP in ALIp and ALIexp associated with IAH at different PEEP levels.** Each symbol is the mean ± SD of eight rats in each group. SD, standard deviation. MAP, Mean Arterial Pressure. ALI, acute lung injury. p, pulmonary. exp, extrapulmonary. IAH, intra-abdominal hypertension. ZEEP, zero end-expiratory pressure. Animals were ventilated with positive end-expiratory pressure (PEEP) of 5, 7, and 10 cmH_2_O.

MAP was maintained stable and above 70 mmHg throughout the experiments (Figure 2). In ALIp, but not in ALIexp, animals in the PEEP10 group required additional fluid (Gelafundin®) administration to maintain MAP >70 mmHg (*P* <0.05) (Table 1).

**Table 1:** Volume of colloid infused to maintain mean arterial pressure above 70 mmHg, as well as arterial oxygen partial pressure and static lung elastance at Baseline-ZEEP

**Etiology**	**PEEP (cmH**_**2**_**O)**	**Volume of colloid (mL)**	**PaO**_**2**_	**Est,L (cmH**_**2**_**O/mL)**
ALIp	5	0.8 (0 to 2.5)	189 ± 93	3.9 ± 0.4
	7	0 (0 to 0.6)	164 ± 71	3.5 ± 0.4
	10	2.3 (1.9 to 2.8)^a^	144 ± 70	3.7 ± 1.3
ALIexp	5	0 (0 to 1.6)	175 ± 91	3.5 ± 0.8
	7	0.3 (0 to 0.6)	125 ± 21	3.3 ± 0.5
	10	1.8 (0.8 to 3.9)	147 ± 58	3.5 ± 0.6

Volume of colloid infused to maintain mean arterial pressure above 70 mmHg, as well as arterial oxygen partial pressure (PaO_2_) and static lung elastance (Est,L) at fraction of inspired oxygen (FiO_2_) = 1.0 and 0.4, respectively. Volume of colloid infused is presented as median (25^th^ to 75^th^ percentile) (eight animals/group), whereas PaO_2_ and Est,L are shown as mean ± SD (eight animals/group). ^a^Significantly different from PEEP7 (*P* <0.05). ALI: acute lung injury; exp: extrapulmonary; p: pulmonary; PEEP: positive end-expiratory pressure; SD, standard deviation; ZEEP: zero end-expiratory pressure.

**Figure 3:**
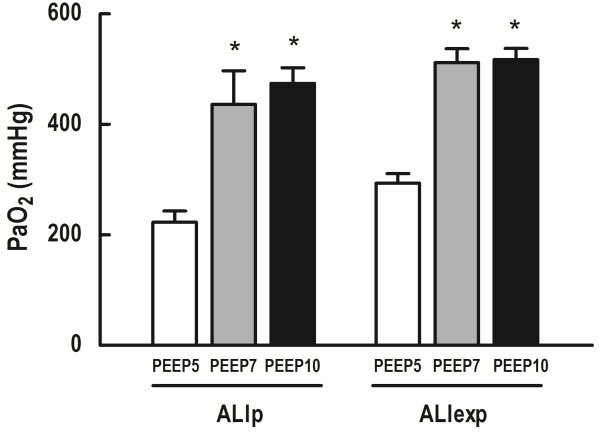
**PaO**_**2 **_**in ALIp and ALIexp associated with IAH at different PEEP levels.** Each bar represents the mean ± SD of eight rats in each group. SD, standard deviation. PaO_2_, arterial oxygen partial pressure. ALI, acute lung injury. p, pulmonary. exp, extrapulmonary. IAH, intra-abdominal hypertension. Animals were ventilated with positive end-expiratory pressure (PEEP) of 5, 7, and 10 cmH_2_O. *Significantly different from PEEP5 (P <0.05).

**Figure 4:**
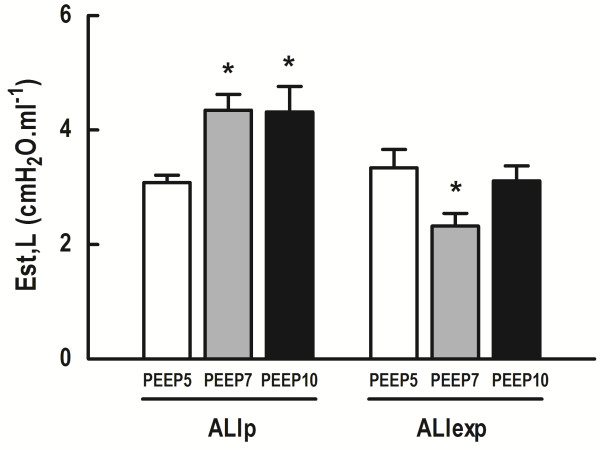
**Est,L at End in ALIp and ALIexp associated with IAH at different PEEP levels.** Each bar represents the mean ± SD of eight rats in each group. SD, standard deviation. Est,L, Static lung elastance. ALI, acute lung injury. p, pulmonary. exp, extrapulmonary. IAH, intra-abdominal hypertension. Animals were ventilated with positive end-expiratory pressure (PEEP) of 5, 7, and 10 cmH_2_O. *Significantly different from PEEP5 (P <0.05).

**Figure 5:**
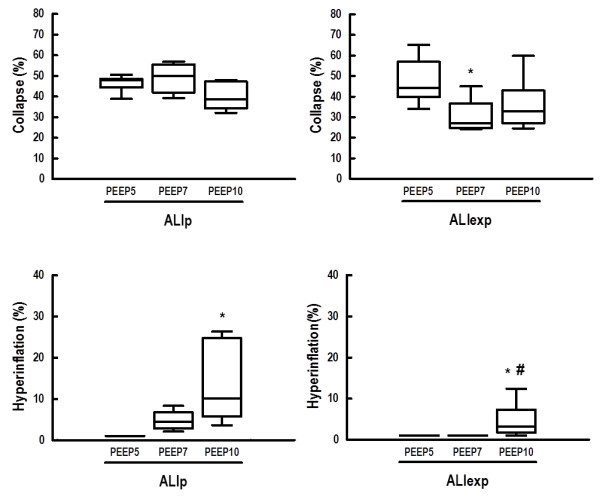
**Alveolar collapse and hyperinflation in ALIp and ALIexp associated with IAH at different PEEP levels.** Box encompasses 25^th^ to 75^th^ percentile, error bars encompass 10^th^ to 90^th^ percentile, and horizontal bar shows the median of eight animals in each group. ALI, acute lung injury. p, pulmonary. exp, extrapulmonary. IAH, intra-abdominal hypertension. Animals were ventilated with positive end-expiratory pressure (PEEP) of 5, 7, and 10 cmH_2_O. *Significantly different from PEEP5 (P <0.05). #Significantly different from PEEP7 (P <0.05).

PaO_2_ and Est,L were comparable among groups at Baseline-ZEEP, regardless of ALI etiology (Table 1). In both ALI groups, at the end of the mechanical ventilation period, PEEP7 and PEEP10 resulted in higher PaO_2_ compared to PEEP5 (Figure 3). In ALIp, PEEP7 and PEEP10 yielded higher Est,L compared to PEEP5. In ALIexp, Est,L was lower in PEEP7 than PEEP5 (Figure 4).ALI groups presented interstitial edema, alveolar collapse and neutrophil infiltration. In ALIp and ALIexp, PEEP10 yielded alveolar hyperinflation (Figure 5). In ALIexp, the fraction area of alveolar collapse was reduced with PEEP7 compared to PEEP5.

**Figure 6:**
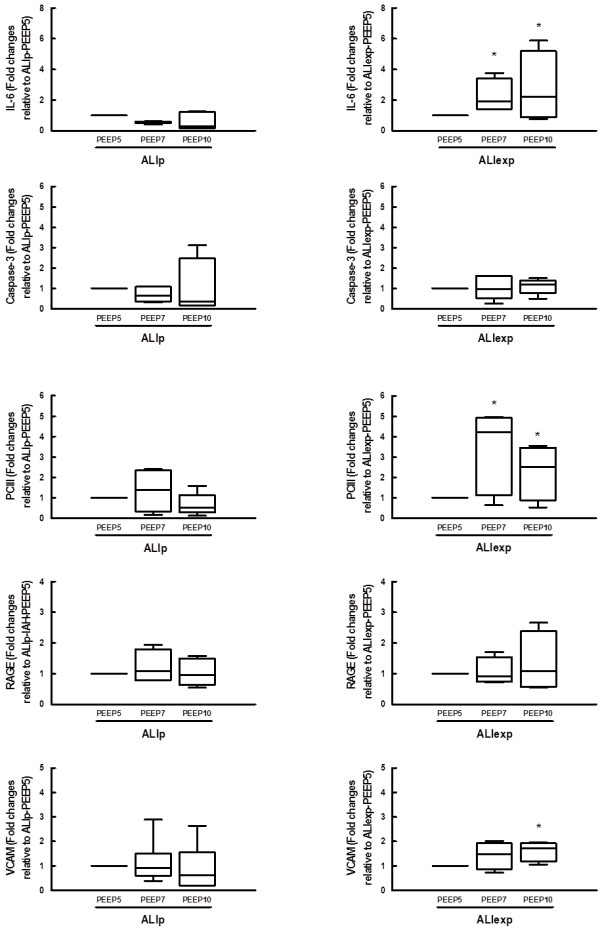
**RT-PCT analysis of biological markers in ALIp and ALIexp with IAH at different PEEP levels.** Biological markers were associated with inflammation (interleukin (IL)-6), apoptosis (caspase-3), fibrogenesis (type III procollagen (PCIII)), damage inflicted on alveolar type I epithelial (receptor for advanced glycation end-products (RAGE)) and endothelial cell (vascular cell adhesion molecule-1 (VCAM-1)). ALI, acute lung injury. p, pulmonary. exp, extrapulmonary. IAH, intra-abdominal hypertension. Animals were ventilated with positive end-expiratory pressure (PEEP) of 5, 7, and 10 cmH_2_O. Relative gene expression was calculated as a ratio of the average gene expression levels compared with the reference gene (36B4) and expressed as fold changes relative to PEEP5. Data are presented as box plot with median lines, 25^th^- and 75^th^-percentile boxes, and 10^th^- and 90^th^- percentile error bars of six animals in each group.*Significantly different from PEEP5 (P <0.05). #Significantly different from PEEP7 (P <0.05).

The semiquantitative analysis of lung and diaphragm electron microscopy is shown in Table 2. The damage of type I and II epithelial cells was more pronounced in ALIp, while endothelial cell and diaphragmatic injury prevailed in ALIexp. In both ALI groups, PEEP10 resulted in greater damage to type I and II epithelial cells. In ALIp, PEEP10 also yielded endothelial cell damage and diaphragmatic injury.The mRNA expression of biological markers associated with inflammation, apoptosis, fibrogenesis and damage inflicted on alveolar type I and II epithelial cells, as well as endothelium are investigated in ALIp and ALIexp (Figure 6). In ALIp, no significant changes were observed in IL-6, caspase-3, PCIII, RAGE and VCAM-1 expressions. In ALIexp, both PEEP7 and PEEP10 increased IL-6 and PCIII expressions, whereas PEEP10 also increased VCAM-1 expression.

**Table 2:** Semiquantitative analysis of lung and diaphragm transmission electron microscopy

		**Lung**			**Diaphragm**		
**Etiology**	**PEEP (cmH**_**2**_**O)**	**Type I epithelial cell**	**Type II epithelial cell**	**Endothelium**	**Myofibril abnormalities**	**Mitochondrial injury**	**Miscellaneous**
ALIp	5	3 (2.75 to 3.25)	2.5 (2 to 3)	2.5 (2 to 3)	3 (2 to 3)	2 (2 to 3)	2.5 (2 to 4)
	7	3 (2 to 3.25)	3 (2.75 to 3)	3 (3 to 4)	3 (2.75 to 4)	2.5 (2 to 3)	3 (2 to 3)
	10	4 (3 to 4)^a^	3 (3.5 to 4)^a^	3.5 (3 to 4)^a^	3 (2.75 to 4)	4 (3 to 4)^a^	3.5 (3 to 4)
ALIexp	5	2 (2 to 2.25)	2 (1.75 to 2)	3 (2.75 to 3.25)	4 (3 to 4)	3 (3 to 4)	4 (3 to 4)
	7	2 (2 to 3)	3 (2.75 to 3)^a^	3 (3 to 4)	3 (3 to 3.25)	3 (3 to 3.25)	3 (3 to 3.25)
	10	3.5 (3 to 4)^ab^	3 (2.75 to 4)^a^	4 (3 to 4)	4 (4 to 4)	4 (3.75 to 4)	4 (3.75 to 4)

The pathologic findings were graded according to a 5-point semiquantitative severity-based scoring system: 0 = normal lung parenchyma, 1 = changes in 1% to 25%, 2 = 26% to 50%, 3 = 51% to 75%, and 4 = 76% to 100% of the examined tissue. Values are the median (25^th^ to 75^th^ percentile) of six animals per group. ^a^Significantly different from PEEP5 (*P* <0.05); ^b^significantly different from PEEP7 (*P* <0.05). ALI: acute lung injury; exp: extrapulmonary; p: pulmonary; PEEP: positive end-expiratory pressure.

## Discussion

The present study focusing on experimental ALIp and ALIexp with IAH revealed that: 1) higher PEEP levels improved oxygenation regardless of ALI etiology; 2) in both ALIp and ALIexp, PEEP10 increased the fraction area of alveolar hyperinflation and epithelial cell damage compared to PEEP5; 3) in ALIp, PEEP7 and PEEP10 increased Est,L compared to PEEP 5, without changes in alveolar collapse, IL-6, caspase-3, PCIII, RAGE, and VCAM-1 expressions, and PEEP10 increased diaphragmatic injury compared to PEEP5; and 4) in ALIexp, while PEEP7 decreased Est,L and the fraction of alveolar collapse compared to PEEP5, both PEEP7 and PEEP10 increased IL-6 and PCIII expressions, as well as type II epithelial cell damage compared to PEEP5. These results suggest that the use of a higher PEEP level in ALI associated with IAH might depend on the underlying ALI etiology. To the best of our knowledge, this is the first study investigating the effects of PEEP on lung morphology, inflammation, apoptosis, fibrogenesis, epithelial and endothelial cell damage, and diaphragmatic injury in ALIp and ALIexp with IAH.

The present models of ALIp and ALIexp have been previously employed [14] and are in accordance with the American Thoracic Society committee recommendations to characterize experimental ALI. IAH was induced by inserting cotton dressings into the abdominal cavity. This model was chosen because it does not result in absorption of liquids or CO_2_ through the peritoneum [9], which could interfere with intravascular volume [15] and inflammation [16], respectively. IAP level was set at 15 mmHg in accordance with the definition of IAH [17].

Animals were ventilated with V_T_ of 6 mL/kg, which is the recommended level during lung-protective mechanical ventilation in ALI [18]. The lowest and highest levels of PEEP were 5 and 10 cmH_2_O, respectively, which are associated with less hemodynamic impairment and fluid requirements in experimental ALI in rats [3,9]. A PEEP of 7 cmH_2_O was used as the intermediate level. A previous study has proposed to set PEEP according to the level of IAP in experimental ALI in pigs [19]. Another recent study has suggested that PEEP higher than IAP maintains functional residual capacity and prevents alveolar collapse [11]. In this line, in the present study, not even the highest PEEP value (10 cmH_2_O = 7.4 mmHg), which was lower than IAP (15 mmHg), was able to prevent alveolar collapse in our animals, independent of ALI etiology. Nevertheless, PEEP values capable of counteracting IAP (approximately 20 cmH_2_O in our study) could lead to hemodynamic instability in these ALI models.

To minimize the influence of confounding factors on lung function and molecular biology data, MAP was maintained stable and above 70 mmHg. The mRNA expression of IL-6 was analyzed because of its role as mediator in the pathogenesis of VALI [20,21], whereas pro-caspase 3 expression was measured since this gene is a surrogate parameter of the final step of apoptosis [22]. Expression of PCIII mRNA was evaluated because this is the first collagen to be remodeled in the presence of lung fibrogenesis [23]; it has also been used as an early marker of lung parenchyma remodeling. RAGE was analyzed because it is abundant in the lung and has been associated with alveolar type I epithelial cell injury [24]. VCAM-1 has been associated with endothelial activation leading to the tethering of inflammatory cells, which may perpetuate the inflammatory process [25].

Current evidence shows that IAH promotes a cephalad shift of the diaphragm [19,26,27], with a reduction in lung volumes and increase in pleural pressure [9,28], causing atelectasis and impaired lung function [29]. In the presence of IAH, higher PEEP is required to counteract the effects of increased IAH, thus minimizing VALI [19]. However, there are controversies regarding the use of PEEP in experimental ALI and clinical ARDS with IAH, which may be related to the variety of experimental models [19,30] and patient populations [31,32].

In this study, PEEP7 and PEEP10 resulted in higher PaO_2_ regardless of ALI etiology. In ALIp, Est,L increased at PEEP10, which may be explained by alveolar hyperinflation. In ALIexp, only PEEP7 was associated with reduced Est,L, which was accompanied by decreased alveolar collapse compared to PEEP5, suggesting partial recruitment of lung units. These different results might be attributed to the predominance of lung consolidation in ALIp, hindering the opening of alveoli, whereas in ALIexp there was a prevalence of recruitable alveoli [8,32]. These findings are in line with those observed in patients with ALIexp with IAH [31]. Conversely, in oleic acid-induced ALI with IAH, Regli *et al*. found no significant changes in Est,L with increased PEEP [19].

In ALIp with IAH, PEEP10 induced greater damage to alveolar type II epithelial cells, which may be caused by the uneven alveolar expansion and, thus, higher local stress in type II epithelial cells that are located in the corners of the alveoli [33]. Moreover, lung inflation distends type I epithelial cells almost twice as much as type II epithelial cells, causing further damage [34]. In previous studies, these ultrastructural changes with higher PEEP were not associated with changes in IL-6, PCIII, caspase-3 and RAGE expressions, suggesting that PEEP7 and PEEP10 were not able to open consolidated alveoli and reduce shear stress [35,36]. Furthermore, in our study, PEEP10 led to additional endothelium cell injury, but no significant changes in VCAM-1 were observed, which may be related to the minimal endothelium cell lesion in ALIp. PEEP10 induced greater damage to the diaphragm in ALIp. It is likely that lung hyperinflation induced by higher PEEP led to distortion of the diaphragm, promoting mitochondrial damage.

In ALIexp, PEEP7 and PEEP10 yielded damage to alveolar type I and II epithelial cells associated with an increase in IL-6 and PCIII. PEEP10 led to an increase in VCAM-1 expression, suggesting that higher PEEP levels increased vascular compression and shear stress across the endothelial barrier, thus promoting greater activation of adhesion molecules [37].

The fact that the increased alveolar epithelial and endothelial cell damage was uncoupled from activation of different biological markers of injury, mainly in ALIp, suggests that those phenomena are linked to different pathways in VALI. The observation that PEEP may induce an increased expression of PCIII and VCAM-1, and not RAGE and caspase-3 could be explained by distinct kinetics of activation of those genes.

### Possible clinical implications

Our results suggest that in ALIp with IAH, changes in Est,L and oxygenation have limited value in setting PEEP to minimize alveolar collapse and VALI. Additionally, our data indicate that in ALIexp associated with IAH, higher PEEP resulted in higher expression of biological markers associated with VALI.

### Limitations

This study has limitations: 1) ALIp and ALIexp were induced by intratracheal and intraperitoneal endotoxin injection; thus, our results cannot be extrapolated to other ALI models with different degrees of severity or human ARDS. Nevertheless, this work represents a step forward in understanding the mechanisms of VALI in experimental ALI with IAH during lung-protective mechanical ventilation; 2) the PEEP levels in the current study, while often used in rats, may not be directly extrapolated to the clinical scenario; 3) we cannot rule out that different results could have been obtained at different IAH and at lower or higher PEEP levels and/or with recruitment maneuvers. Recruitment maneuvers were not applied associated with PEEP to avoid confounding effects concerning the different biological impact on lung parenchyma in each ALI group [8,38]; 4) the mediators were measured in lung tissue but not in the blood; 5) the observation time was relatively short (one hour) compared to previous studies which ventilated rats for four [39] or six hours [40], precluding changes in the protein levels of all these biological markers. However, in order to keep alive small animals with ALI associated with IAH for six hours, it is necessary to use higher amounts of fluids, sometimes vasoactive drugs (for example, noradrenaline) to maintain MAP higher than 60 mmHg, and bicarbonate due to intense metabolic acidosis. All these therapeutic strategies interfere with individual gene activation. Moreover, each protein mediator was synthetized at different time points and at four hours no protein synthesis occurs. Thus, as a primary study design, even though one hour duration represents a short study time, we are able to evaluate the gene activation induced by the PEEP in different ALI etiologies associated with IAH without the interference of necessary therapies to keep the animal alive. This experimental study may be regarded as the first step to help other experimental and clinical studies start evaluating the effects of different PEEP levels in ALI associated with IAH; and 6) it is difficult at the present stage to understand fully the mechanisms associated with individual gene activation, and thus another study design would be required.

## Conclusions

In these experimental models of ALI, higher PEEP should be cautiously used in the presence of IAH, mainly in ALIexp.

## Key messages

● In the current models of ALIp and ALIexp with IAH, higher PEEP improved oxygenation, but yielded epithelial cell damage and alveolar hyperinflation, without avoiding collapse.

● In ALIp with IAH, higher PEEP led to endothelial cell and diaphragm damage.

● In ALIexp with IAH, higher PEEP worsened markers of lung inflammation, fibrogenesis, and endothelial cell damage, whereas moderate PEEP was able to reduce static lung elastance, alveolar collapse, and inflammation, and thus lung protection.

● In ALI, higher PEEP should be cautiously used in the presence of IAH, mainly in ALIexp.

## Abbreviations

ALIexp: extrapulmonary acute lung injury; ALIp: pulmonary acute lung injury; Est,L: static lung elastance; FiO_2_: fraction of inspired oxygen; IAH: intra-abdominal hypertension; IAP: intra-abdominal pressure; IL-6: interleukin-6; I:E: inspiratory:expiratory ratio; LPS: lipopolysaccharide; MAP: mean arterial pressure; PaO_2_: partial pressure of arterial oxygen; P,aw: airway pressure; PCIII: type III procollagen; PEEP: positive end-expiratory pressure; PEEP5: PEEP of 5 cmH_2_O; PEEP7: PEEP of 7 cmH_2_O; PEEP10: PEEP of 10 cmH_2_O; Pes: esophageal pressure; P,L: transpulmonary pressure; RAGE: receptor for advanced glycation end products; TEM: transmission electron microscopy; VALI: ventilator-associated lung injury; VCAM-1: vascular cell adhesion molecule-1; V_T_: tidal volume; ZEEP: zero end-expiratory pressure.

## Competing interests

The authors declare they have no compting interests.

## Authors’ contributions

CLS participated in the design of the study, carried out the experiments, performed data analyses and drafted the manuscript; LM, RSS and CSS contributed to the study design, carried out the experiments, performed data analyses and wrote the manuscript; JDS provided expert assistance during experiments, analyzed mechanical data and helped draft the manuscript; MMM carried out the molecular biology analyses and contributed to the manuscript; VLC performed the histological analyses and helped draft the manuscript; MGA, AS, PLS and PP contributed to the study design, supervised the entire project and helped write the manuscript; CSNBG and PRMR contributed to the study design, supervised the experimental work and statistical analysis, wrote the manuscript and supervised the entire project. All authors read and approved the final manuscript.

## Supplementary Material

Additional file 1: Table S1 Containing the used target genes primer sequences for RT-PCR. Click here for file

## References

[B1] Amato MB Barbas CS Medeiros DM Magaldi RB Schettino GP Lorenzi-Filho G Kairalla RA Deheinzelin D Munoz C Oliveira R Takagaki TY Carvalho CR Effect of a protective-ventilation strategy on mortality in the acute respiratory distress syndrome N Engl J Med 1998 338 347 354 9449727 10.1056/NEJM199802053380602

[B2] Hoelz C Negri EM Lichtenfels AJ Concecao GM Barbas CS Saldiva PH Capelozzi VL Morphometric differences in pulmonary lesions in primary and secondary ARDS, a preliminary study in autopsies Pathol Res Pract 2001 197 521 530 11518044

[B3] Passaro CP Silva PL Rzezinski AF Abrantes S Santiago VR Nardelli L Santos RS Barbosa CM Morales MM Zin WA Amato MB Capelozzi VL Pelosi P Rocco PR Pulmonary lesion induced by low and high positive end-expiratory pressure levels during protective ventilation in experimental acute lung injury Crit Care Med 2009 37 1011 1017 19237911 10.1097/CCM.0b013e3181962d85

[B4] Carvalho AR Spieth PM Pelosi P Vidal Melo MF Koch T Jandre FC Giannella-Neto A de Abreu MG Ability of dynamic airway pressure curve profile and elastance for positive end-expiratory pressure titration Intensive Care Med 2008 34 2291 2299 18825365 10.1007/s00134-008-1301-7 PMC3177558

[B5] Nieszkowska A Lu Q Vieira S Elman M Fetita C Rouby JJ Incidence and regional distribution of lung overinflation during mechanical ventilation with positive end-expiratory pressure Crit Care Med 2004 32 1496 1503 15241094 10.1097/01.ccm.0000130170.88512.07

[B6] Cressoni M Cadringher P Chiurazzi C Amini M Gallazzi E Marino A Brioni M Carlesso E Chiumello D Quintel M Bugedo G Gattinoni L Lung inhomogeneity in patients with acute respiratory distress syndrome Am J Respir Crit Care Med 2014 189 149 158 24261322 10.1164/rccm.201308-1567OC

[B7] Pelosi P Rocco PR Effects of mechanical ventilation on the extracellular matrix Intensive Care Med 2008 34 631 639 18264691 10.1007/s00134-007-0964-9

[B8] Riva DR Oliveira MB Rzezinski AF Rangel G Capelozzi VL Zin WA Morales MM Pelosi P Rocco PR Recruitment maneuver in pulmonary and extrapulmonary experimental acute lung injury Crit Care Med 2008 36 1900 1908 18496360 10.1097/CCM.0b013e3181760e5d

[B9] Santos CL Moraes L Santos RS Oliveira MG Silva JD Maron-Gutierrez T Ornellas DS Morales MM Capelozzi VL Jamel N Pelosi P Rocco PR Garcia CS Effects of different tidal volumes in pulmonary and extrapulmonary lung injury with or without intraabdominal hypertension Intensive Care Med 2012 38 499 508 22234736 10.1007/s00134-011-2451-6

[B10] Vazquez de Anda GF Gommers D Verbrugge SJ De Jaegere A Lachmann B Mechanical ventilation with high positive end-expiratory pressure and small driving pressure amplitude is as effective as high-frequency oscillatory ventilation to preserve the function of exogenous surfactant in lung-lavaged rats Crit Care Med 2000 28 2921 2925 10966272 10.1097/00003246-200008000-00039

[B11] Silva PL Moraes L Santos RS Samary C Ramos MB Santos CL Morales MM Capelozzi VL Garcia CS de Abreu MG Pelosi P Marini JJ Rocco PR Recruitment maneuvers modulate epithelial and endothelial cell response according to acute lung injury etiology Crit Care Med 2013 41 e256 e265 23887231 10.1097/CCM.0b013e31828a3c13

[B12] Baydur A Behrakis PK Zin WA Jaeger M Milic-Emili J A simple method for assessing the validity of the esophageal balloon technique Am Rev Respir Dis 1982 126 788 791 7149443 10.1164/arrd.1982.126.5.788

[B13] Cruz-Orive LM Weibel ER Recent stereological methods for cell biology: a brief survey Am J Physiol 1990 258 L148 L156 2185653 10.1152/ajplung.1990.258.4.L148

[B14] Matute-Bello G Downey G Moore BB Groshong SD Matthay MA Slutsky AS Kuebler WM Acute Lung Injury in Animals Study Group An official American Thoracic Society workshop report: features and measurements of experimental acute lung injury in animals Am J Respir Cell Mol Biol 2011 44 725 738 21531958 10.1165/rcmb.2009-0210ST PMC7328339

[B15] Mutoh T Lamm WJ Embree LJ Hildebrandt J Albert RK Volume infusion produces abdominal distension, lung compression, and chest wall stiffening in pigs J Appl Physiol 1992 72 575 582 1559935 10.1152/jappl.1992.72.2.575

[B16] Kopernik G Avinoach E Grossman Y Levy R Yulzari R Rogachev B Douvdevani A The effect of a high partial pressure of carbon dioxide environment on metabolism and immune functions of human peritoneal cells-relevance to carbon dioxide pneumoperitoneum Am J Obstet Gynecol 1998 179 1503 1510 9855588 10.1016/s0002-9378(98)70016-x

[B17] Kirkpatrick AW Roberts DJ De Waele J Jaeschke R Malbrain ML De Keulenaer B Duchesne J Bjorck M Leppaniemi A Ejike JC Sugrue M Cheatham M Ivatury R Ball CG Reintam Blaser A Regli A Balogh ZJ D’Amours S Debergh D Kaplan M Kimball E Olvera C Pediatric Guidelines Sub-Committee for the World Society of the Abdominal Compartment Syndrome Intra-abdominal hypertension and the abdominal compartment syndrome: updated consensus definitions and clinical practice guidelines from the World Society of the Abdominal Compartment Syndrome Intensive Care Med 2013 39 1190 1206 23673399 10.1007/s00134-013-2906-z PMC3680657

[B18] Putensen C Theuerkauf N Zinserling J Wrigge H Pelosi P Meta-analysis: ventilation strategies and outcomes of the acute respiratory distress syndrome and acute lung injury Ann Intern Med 2009 151 566 576 19841457 10.7326/0003-4819-151-8-200910200-00011

[B19] Regli A Mahendran R Fysh ET Roberts B Noffsinger B De Keulenaer BL Singh B van Heerden PV Matching positive end-expiratory pressure to intra-abdominal pressure improves oxygenation in a porcine sick lung model of intra-abdominal hypertension Crit Care 2012 16 R208 23098278 10.1186/cc11840 PMC3682312

[B20] Ware LB Pathophysiology of acute lung injury and the acute respiratory distress syndrome Semin Respir Crit Care Med 2006 27 337 349 16909368 10.1055/s-2006-948288

[B21] Haitsma JJ Uhlig S Verbrugge SJ Goggel R Poelma DL Lachmann B Injurious ventilation strategies cause systemic release of IL-6 and MIP-2 in rats in vivo Clin Physiol Funct Imaging 2003 23 349 353 14617266 10.1046/j.1475-0961.2003.00518.x

[B22] Slee EA Harte MT Kluck RM Wolf BB Casiano CA Newmeyer DD Wang HG Reed JC Nicholson DW Alnemri ES Green DR Martin SJ Ordering the cytochrome c-initiated caspase cascade: hierarchical activation of caspases-2, −3, −6, −7, −8, and −10 in a caspase-9-dependent manner J Cell Biol 1999 144 281 292 9922454 10.1083/jcb.144.2.281 PMC2132895

[B23] Raghu G Striker LJ Hudson LD Striker GE Extracellular matrix in normal and fibrotic human lungs Am Rev Respir Dis 1985 131 281 289 3882034 10.1164/arrd.1985.131.2.281

[B24] Uchida T Shirasawa M Ware LB Kojima K Hata Y Makita K Mednick G Matthay ZA Matthay MA Receptor for advanced glycation end-products is a marker of type I cell injury in acute lung injury Am J Respir Crit Care Med 2006 173 1008 1015 16456142 10.1164/rccm.200509-1477OC PMC2662912

[B25] Perl M Lomas-Neira J Venet F Chung CS Ayala A Pathogenesis of indirect (secondary) acute lung injury Expert Rev Respir Med 2011 5 115 126 21348592 10.1586/ers.10.92 PMC3108849

[B26] Quintel M Pelosi P Caironi P Meinhardt JP Luecke T Herrmann P Taccone P Rylander C Valenza F Carlesso E Gattinoni L An increase of abdominal pressure increases pulmonary edema in oleic acid-induced lung injury Am J Respir Crit Care Med 2004 169 534 541 14670801 10.1164/rccm.200209-1060OC

[B27] Regli A Hockings LE Musk GC Roberts B Noffsinger B Singh B van Heerden PV Commonly applied positive end-expiratory pressures do not prevent functional residual capacity decline in the setting of intra-abdominal hypertension: a pig model Crit Care 2010 14 R128 20598125 10.1186/cc9095 PMC2945091

[B28] Pelosi P Luecke T Rocco PR Chest wall mechanics and abdominal pressure during general anaesthesia in normal and obese individuals and in acute lung injury Curr Opin Crit Care 2011 17 72 79 21150585 10.1097/MCC.0b013e3283427213

[B29] Ranieri VM Brienza N Santostasi S Puntillo F Mascia L Vitale N Giuliani R Memeo V Bruno F Fiore T Brienza A Impairment of lung and chest wall mechanics in patients with acute respiratory distress syndrome: role of abdominal distension Am J Respir Crit Care Med 1997 156 1082 1091 9351606 10.1164/ajrccm.156.4.97-01052

[B30] Runck H Schumann S Tacke S Haberstroh J Guttmann J Effects of intra-abdominal pressure on respiratory system mechanics in mechanically ventilated rats Respir Physiol Neurobiol 2012 180 204 210 22138515 10.1016/j.resp.2011.11.007

[B31] Krebs J Pelosi P Tsagogiorgas C Alb M Luecke T Effects of positive end-expiratory pressure on respiratory function and hemodynamics in patients with acute respiratory failure with and without intra-abdominal hypertension: a pilot study Crit Care 2009 13 R160 19804634 10.1186/cc8118 PMC2784387

[B32] Gattinoni L Pelosi P Suter PM Pedoto A Vercesi P Lissoni A Acute respiratory distress syndrome caused by pulmonary and extrapulmonary disease. Different syndromes? Am J Respir Crit Care Med 1998 158 3 11 9655699 10.1164/ajrccm.158.1.9708031

[B33] Roan E Waters CM What do we know about mechanical strain in lung alveoli? Am J Physiol Lung Cell Mol Physiol 2011 301 L625 L635 21873445 10.1152/ajplung.00105.2011 PMC3213982

[B34] Perlman CE Bhattacharya J Alveolar expansion imaged by optical sectioning microscopy J Appl Physiol (1985) 2007 103 1037 1044 17585045 10.1152/japplphysiol.00160.2007

[B35] Caironi P Cressoni M Chiumello D Ranieri M Quintel M Russo SG Cornejo R Bugedo G Carlesso E Russo R Caspani L Gattinoni L Lung opening and closing during ventilation of acute respiratory distress syndrome Am J Respir Crit Care Med 2010 181 578 586 19910610 10.1164/rccm.200905-0787OC

[B36] Wellman TJ Winkler T Costa EL Musch G Harris RS Venegas JG Vidal Melo MF Effect of regional lung inflation on ventilation heterogeneity at different length scales during mechanical ventilation of normal sheep lungs J Appl Physiol 2012 113 947 957 22678958 10.1152/japplphysiol.01631.2011 PMC3472483

[B37] Silva PL Cruz FF Fujisaki LC Oliveira GP Samary CS Ornellas DS Maron-Gutierrez T Rocha NN Goldenberg R Garcia CS Morales MM Capelozzi VL Gama de Abreu M Pelosi P Rocco PR Hypervolemia induces and potentiates lung damage after recruitment maneuver in a model of sepsis-induced acute lung injury Crit Care 2010 14 R114 20546573 10.1186/cc9063 PMC2911760

[B38] Silva PL Moraes L Santos RS Samary C Ornellas DS Maron-Gutierrez T Morales MM Saddy F Capelozzi VL Pelosi P Marini JJ Gama de Abreu M Rocco PR Impact of pressure profile and duration of recruitment maneuvers on morphofunctional and biochemical variables in experimental lung injury Crit Care Med 2011 39 1074 1081 21263326 10.1097/CCM.0b013e318206d69a

[B39] Wosten-van Asperen RM Lutter R Specht PA van Woensel JB van der Loos CM Florquin S Lachmann B Bos AP Ventilator-induced inflammatory response in lipopolysaccharide-exposed rat lung is mediated by angiotensin-converting enzyme Am J Pathol 2010 176 2219 2227 20304959 10.2353/ajpath.2010.090565 PMC2861087

[B40] Krebs J Pelosi P Tsagogiorgas C Zoeller L Rocco PR Yard B Luecke T Open lung approach associated with high-frequency oscillatory or low tidal volume mechanical ventilation improves respiratory function and minimizes lung injury in healthy and injured rats Crit Care 2010 14 R183 20946631 10.1186/cc9291 PMC3219289

