# Supplementary material for: The biological effects of higher and lower positive end-expiratory pressure in pulmonary and extrapulmonary acute lung injury with intra-abdominal hypertension
Source: Crit Care. 2014 Jun 13;18(3):R121. doi: 10.1186/cc13920 (PMC4095606; doi:10.1186/cc13920)
Supplement: Additional file 1: Table S1 — Containing the used target genes primer sequences for RT-PCR. [file cc13920-S1.pdf]

Additional File 1

Table S1. Target genes primer sequence

	**Sense**	**Antisense**
IL-6	5'-CTCCGCAAGAGACTTCCAG-3'	5'-CTCCTCTCCGGACTTGTG A-3'
Caspase-3	5'-GGCCGACTTCCTGTATGC-3'	5'-GCGCAAAGTGACTGGATG-3'
PCIII	5'-ACCTGGACCACAAGGACAC-3'	5'-TGGACCCATTTCACCTTTC-3'
RAGE	5'-TGAACTCACAGCCAATGTCC-3'	5'-ACAACTGTCCCTTTGCCATC-3'
VCAM-1	5'-TGCACGGTCCCTAATGTGTA-3'	5'-TGCCAATTTCCTCCCTTAAA-3'
36B4	5'-AATCCTGAGCGATGTGCAG-3'	5'-GCTGCCATTGTCAAACAC-3'

Primers used in experiments. IL-6: interleukin, PCIII: type III pro-collagen, RAGE: receptor for advanced glycation end-products, and VCAM-1: vascular cell adhesion molecule-1.
